# 760. Multidisciplinary Education in Substance Use Disorder Centers Improves HIV Prevention Knowledge and Practice: BRIDGE PrEP: Building Bridges to Reach People Who Inject Drugs with the Goal of Employing PrEP for HIV Prevention

**DOI:** 10.1093/ofid/ofad500.821

**Published:** 2023-11-27

**Authors:** Amanda Glazar, Becky K Carney, Mara S Simpson, Susan Daniels, Gregory S Felzien

**Affiliations:** Integritas Communications, Evergreen, Colorado; Integritas Communications, Evergreen, Colorado; Integritas Commuincations, MONUMENT, Colorado; Integritas Communications, Evergreen, Colorado; Positive Impact Health Centers, Inc., Lilburn, Georgia

## Abstract

**Background:**

Around 10% of new HIV infections in the US are attributed to injection drug use or male-to-male contact and injected drug use; syringe sharing is the second riskiest behavior for contracting HIV. For many, substance use disorder (SUD) treatment is the primary point of access for receiving HIV-related services. A 2021 survey showed gaps in HIV prevention knowledge and practice among SUD center staff but also interest in education (Fig 1). The BRIDGE PrEP program is a grassroots initiative designed to educate and motivate SUD center staff to increase their clients’ awareness of and access to HIV preexposure prophylaxis (PrEP) services.

**Figure:**
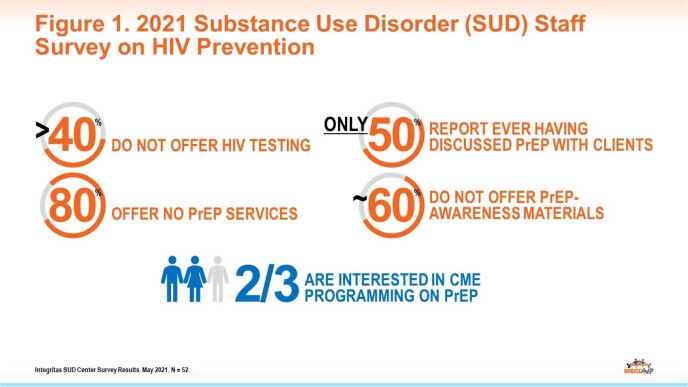

**Methods:**

Ten live continuing medical education (CME) meetings supported by an educational grant from Gilead Sciences, Inc were conducted at SUD centers between August 2022 and May 2023. The 1-hour meetings provided didactic information, real-world insights, faculty-learner interactions, local networking, and take-away print and online staff/patient-education resources (Fig 2). Program goals include:1.Increase HIV testing of people who inject drugs 2. Educate SUD center staff regarding PrEP harm-reduction practices and 3.Build linkages between SUD staff, PrEP providers, and HIV-care networks within local/regional systems. Moore’s Level 1-5 (self-reported) outcomes (Fig 3) were collected via pre-, post-, and 6-week follow-up surveys evaluating knowledge and self-reported frequencies of recommended HIV prevention practice behaviors.

**Figure:**
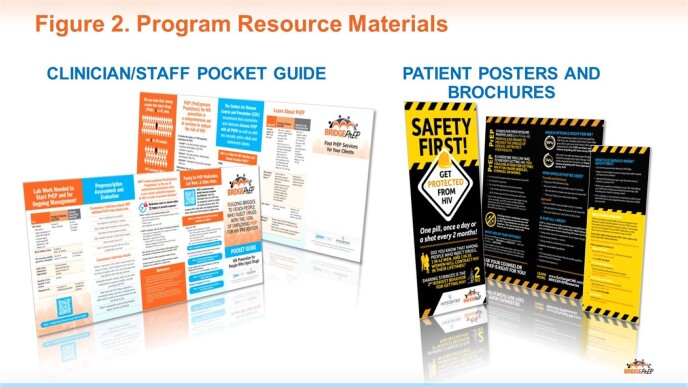

**Figure:**
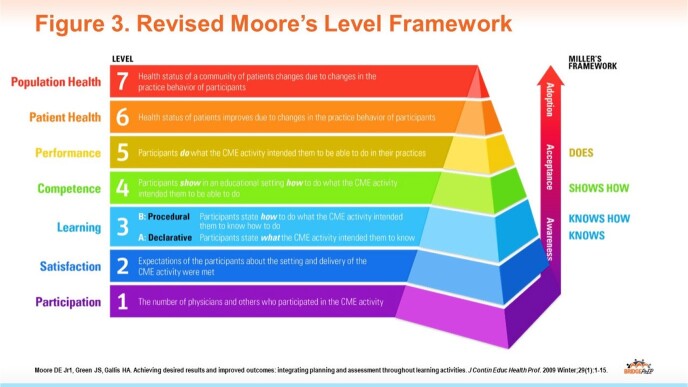

**Results:**

To date, there have been 154 participants representing a diverse multidisciplinary learner group extending beyond the clinician-only audience typical of many CME programs (Fig 4). Increases in knowledge were demonstrated across topic areas (Fig 5), and participants reported intent to improve HIV prevention-related practices following the activity (Fig 6). To date, follow-up surveys report specific actions taken on individual and system levels to improve HIV screening and prevention (Fig 7).

**Figure:**
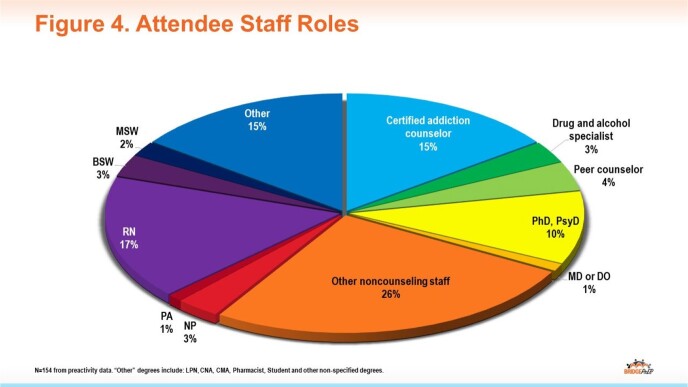

**Figure:**
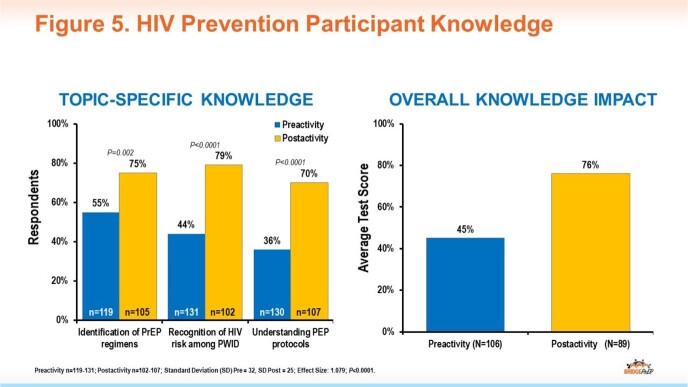

**Figure:**
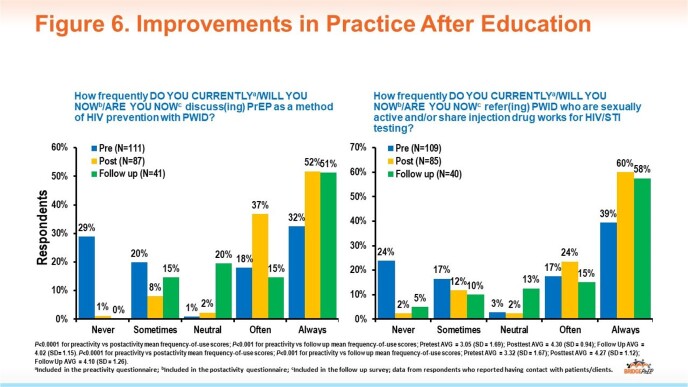

**Conclusion:**

Positive outcomes related to HIV prevention knowledge and practice among SUD center staff have the potential to impact many at-risk individuals. Additionally, follow-up surveys suggest that improved awareness is prompting collaboration and system-level changes driven by participants (Fig 8).

**Figure:**
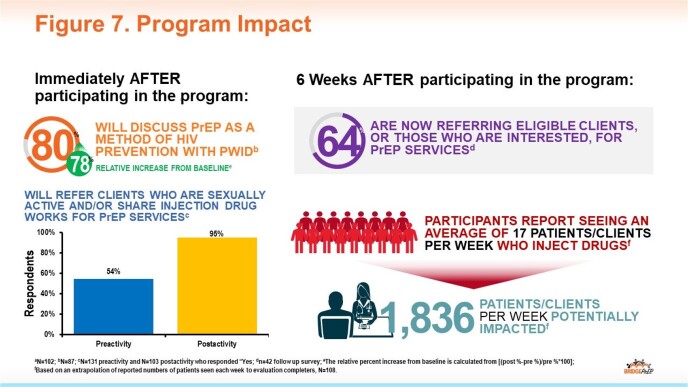

**Figure:**
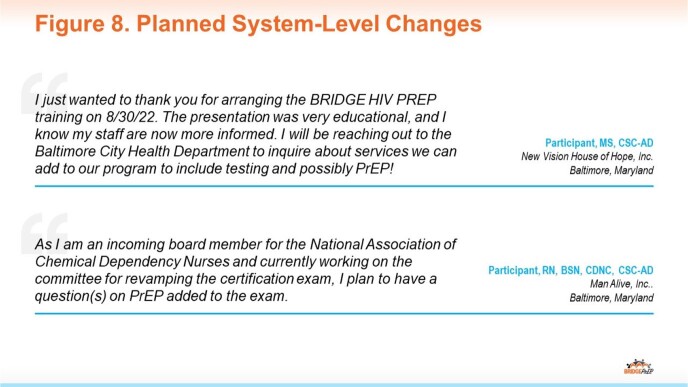

**Disclosures:**

**All Authors**: No reported disclosures

